# Extracts and Gleanings

**Published:** 1872-03

**Authors:** 


### PART II.

### Abridged Extracts and Gleanings from our Exchanges

*MULTUM IN PARVO.*

ADDISON'S DISEASE.—Dr. Finlayson reports in the *Glasgow Medical Journal* for August, 1871, a case of this disease. The following is a catalogue of the symptoms in the order of their appearance: Languor, feebleness, and impaired appetite; discoloration of the face, neck, and hands; pains in the back; profound asthænia; occasional vomiting; discoloration of the mucous membrane of the mouth; hiccough; discoloration of fresh portions of the skin; giddiness; mistiness of eyesight; slight diarrhœa; fever; delirium; tremors; unconsciousness, and death.

FURUNCULOUS ABSCESS IN THE EXTERNAL MEATUS.—These abscesses are most exquisitely painful, slow in development, and form a core which is discharged with difficulty. There is also a plugging up of the canal by a swelling of its tissues. The most successful treatment has been the application of moist warmth to the parts and a free incision into the abscess as soon as its presenting point could be determined.—*Dr. L. Srumbull.*

CROTON-CHLORAL.—Dr. Oscar Liebreich has discovered another new substance for anæsthetic purposes. Dr. Julius Althaus (who reports these investigations in the *Medical Times and Gazette*) considers, therefore, that croton-chloral promises to produce all the good effects of hydrate of chloral without any drawback being attached to its judicious use. Its apparently specific effects on the fifth pair of cerebral nerves makes us indulge the hope that it may perhaps be found useful in that intractable affection—true tic doloureux, or epileptiform neuralgia of the face.—*Med. & Surg. Reporter.*

IRON IN UTERINE INJECTIONS.—In a discussion on uterine injections, at a late meeting of the New York Obstetrical Society (*Am. Journal Obstetrics*), Dr. Noeggerath believed the subsulphate of iron less dangerous than the sesquichloride, from the use of which he once occasioned a metro-pritonitis.

Management of Epilepsy.—Dr. Brown-Sequard recommends, in the treatment of epilepsy, the following combination of the bromides of ammonium and potassium :

| | |
|---|---|
| R.—Potassi iodidi, . . . . | dr. j. |
| Potass. bicarb, . . . . | scr. ij. |
| Potassi bromidi, . . . | oz. j. |
| Ammonii bromidi, . . . | dr. iiss. |
| Inf. columbæ, . . . . | oz. vj. |

S.—A teaspoonful before each of the three meals, and three teaspoonfuls at bedtime, with a little water.

Dr. Robert Bartholow's (Fisk Fund Prize Essay) plan of treatment consists in giving a powder, containing two scruples of bromide of potassium dissolved in water, three times a day, and after the cessation of the paroxysms a drachm dose at bedtime only. It is now well known that a patient cannot omit his dose for a single day without danger of having the attacks return, and he cannot be considered exempt until he has passed two years without a convulsive seizure.

To prevent the development of bromism Brown-Sequard is in the habit of combining arsenic with the bromide of potassium. Since using this combination, he has not observed so much the debility caused by its prolonged administration. The use of iron, strychnia, the hypophosphites, is also indicated to maintain the health of epileptics during a course of bromide of potassium. The hygienical means consists of abundant food, wine, out-door employment, and a careful regulation of the moral life.—*Medical Record.*

Strychnia in Hypodermic Medication.—Dr. R. A. Vance, of N. Y. (*Med. World*), says that the solution of strychnia, for hypodermic medication, which his experience has taught him to consider best is one containing a grain of the drug to one drachm of water ; the solution being effected by the addition of a small quantity of dilute acetic acid. His formula is the following:

| | |
|---|---|
| R.—Strychniœ sulph., . . . | gr. i. |
| Acid. acet. dil., . . . . | m. i. |
| Aquœ ad . . . . . | dr. i.—M. |

S.—Ft. sol.

[*Medical Record.*

THE CURE OF CANCER BY ELECTROLYSIS.—Dr. Neftel (*Medical Record*), presented sections of carcinomatus deposit removed post mortem from a lady who had died in consequence of mammary cancer. About two years ago she noticed a hard and painful lump in right mamma. This increased, and with the pain extended to the axilla. These masses were removed by operation. Soon after the operation she had an attack of pneumonia, from which she did not recover until the lapse of several months. In the meantime the wound cicatrized, but the pain still continued, and extended down the arm of that side, making it almost useless. After several months she felt that the cicatrix became indurated, and from these there seemed to be a string of smaller lumps, which aroused the suspicion in Dr. N.'s mind that the disease had translated itself to some internal organ; she then insisted upon being treated by electrolysis, and the treatment was pursued in conjunction with Dr. Bailey, of Albany. To the surprise of Dr. N. not only did the secondary tumors disappear, but the patient improved in general health. So marked was this latter effect that Dr. N. was inclined to believe that he had been mistaken in his diagnosis of internal metastasis. After several months tumors again showed themselves in the same locality; these were treated, and likewise disappeared. Finally, the cervical glands became affected, and she began to suffer from asthmatic attacks in consequence of pressure upon the pneumogastric; these were succeeded by an attack of pleurisy, due to cancerous exudation, and she finally died delirious. At the autopsy the liver, lungs, and cervical glands were found infiltrated with cancerous material.

In speaking of the effects of electrolytic treatment upon cancer, Dr. N. stated that he had reason to believe it would always be successful, if employed before the disease become constitutional.

CARDED WOOL IN THE TREATMENT OF WOUNDS.—Dr. Walker (*Practitioner*), has tried various materials for dressing wounds, but gives a decided preference to carded wool, which is known as marine lint. He says of its use:

Take an amputation of arm or leg. After the operation, my plan of treatment is as follows: Sponge out the wound thoroughly with chloride of zinc dr. ss to oz. j of water, then let all applications be quite dry for the first two or three days; as soon as there is the least secretion of pus, take a Higginson's syringe, and wash out the flaps with a strong solution of Condy's fluid, say oz. j to oz. x of water; apply strapping, and cover the wound with a small piece of lint wetted with a lotion of dr. ij carbolic acid dr. ij liq. potassæ to six ounces of water; cover with a piece of guttapercha tissue, and continue until healed, surrounding the edges or flaps with a little pad of carded lint.

Take another every-day case—an ulcer of the leg. If it is a large flabby ulcer, secreting a large quantity of pus, nothing is so good for compressing the granulations and checking the secretion of pus as the marine wool; but after it has fulfilled its object, a change of treatment, by the application of either a little dry precipitated chalk, zinc-ointment, or zinc-lotion, will heal it in a few days, with rest and bandaging.

Treatment of Bubo.—Dr. W. H. McNamara, objecting to the usual method of opening an acute bubo, says:

"I have for some time practised the opening of bubo by means of potassa fusa. The bubo is covered over with several layers of sticking plaster, in which a hole, *half* the size of the intended opening, had been previously made; potassa fusa is now rubbed on the exposed skin, through the hole; sticking-plaster is then put over the hole, and a full dose of opium given. Very little irritation is produced, and the patient expresses his satisfaction that the knife has not been used. In a short time, a black *eschar* is formed, which is removed in a few days by poultices, and a healthy ulcer, easily healed by the usual treatment, is the result.

Chronic bubo, when opened by the knife, is even more troublesome than the acute disease, on account of the well known thin blue skin which surrounds the opening and prevents healthy action. It is usually recommended to get rid of this by means of potassa fusa, or some other caustic, but, if potassa fusa is used, as described above, in opening the bubo, this unhealthy skin does not appear.

In some cases of chronic, painless, indolent bubo, small suppurating centres occur without interfering much with the vitality of the skin which covers them. In these cases an apparatus, which lets out the matter without letting in the air, has been employed with very good effect.

As instruments are not always at hand, it occurred to me that by running a lancet under the skin, commencing a few lines from the seat of suppuration into the abscess, squeezing out the matter, and applying a pad with a bandage, a similar result would ensue. I have practised this method in several instances, with the effect of curing the patient in a few days.

Arsenic in Menorrhagia and Leucorrhœa.—(*The Doctor.*) Before the British Medical Association Dr. J. H. Aveling read a paper on this subject. He believes that this remedy has not received from the profession the attention it deserves. Dr. Henry Hunt used it successfully in uterine disorders, and published his experience in 1838. Dr. Aveling has employed it in cases of menorrhagia for twelve years with great advantage. Besides improving nutrition, respiration and secretion, he finds it to have a powerful decongestive action upon all mucous membranes. He administers small doses of arsenic either in solution or in granules, and continues them for weeks or months, as the necessities of the case may require. He believes the forms of menorrhagia and leucorrhœa most amenable to the arsenic-treatment are those which have their origin in a hyperæmic condition of the uterus, which state the remedy cures by acting as a tonic and stimulant upon the vaso-motor nerves, causing the capillaries to contract and expel the superabundant blood.

Treatment of Diptheria with Carbolic Acid.—*Dehr. Klinik.*, 26, 1871.)—Dr. Helfer employs carbolic acid in the proportions of 1 to 200 as gargle and injection, in the proportion of 1 to 50 for inhalation, and in concentrated solution with the camel's-hair pencil. He had treated altogether sixteen cases of diptheria with carbolic acid; the majority of these cases were diptheritis scarlatinosa, a few most probably angina diptheritica sine exanthemata, and, as far as can be gathered from the reports of the cases, at the most, two or three were cases of idiopathic diptheritis. The results which Dr. Helfer reports are of a nature to encourage experiments with the carbolic acid, and he refers also to Prof. Henwig and Dr. Weikert, who had obtained excellent results from the same treatment. In two cases also of pure croup, and in one of diphtheritic croup, the employment of carbolic acid resulted in a cure.—(See *Med.-Chir. Records*, No. 161, 1871.)—*N. Y. Medicnl Journal.*

Taraxacum Coffee.—Jno. R. Jackson, A. L. S., (*Phar. Jour. & Trans.*), writing of taraxacum, says:

"Taraxacum roots are used in a variety of ways in India; one useful form is that of a paste, which is made by pounding the fresh roots, putting the mass into tins or jars and gently baking or heating in an oven; when cool, the paste is ready for use, and can be kept for a long time. To prepare dandelion-coffee, the roots are washed, dried in the sun and cut up into small pieces, after which they are roasted in a similar manner to true coffee: they are then ground, and to every nine ounces of coffee one ounce of pounded dandelion root may be added; these proportions make an excellent and useful beverage. The use of this coffee in India has been much recommended.

Lieutenant Pegson, in a communication to the Agri-horticultural Society of India, advocating the more general cultivation and use of the dandelion, says, "Medical men admit the value of this preparation, and I know several gentlemen in India who are, by their own admission, kept alive by the daily use of taraxacum-coffee. It is fairly entitled to be called a specific for the cure of torpid liver, a complaint from which the majority of Europeans suffer."

As the dandelion is cultivated in this country, why not test the value of "taraxacum-coffee" in the "torpid livers" of our Southern climate?—Eds.

Cardialgia (Heart-burn).—Geo. Archbold, D. SC., (*Phar. Jour. & Trans.*), has tested the beneficial action of liq. magnesiæ bisulphites for butyric acid fermentation. He remarks:

"Now, as bisulphites have the power of preventing this fermentation, they are well worthy the attention of the profession, but the great drawback is that the chief bisulphite manufactures are those of lime, soda and potash, these being objectionable, as they tend to injure the coats of the stomach. To remedy this failing, the thought at once suggested itself to me that a bisulphite of magnesia might be prepared; and magnesia being free from these objections, it may prove a valuable remedy, and is worth notice. I have not seen or heard anything of the preparation previous to my making it. I therefore give a brief outline of the process I adopt, and hope to enter more fully into the subject at a future time. I first treat magnesia carbonate with B. P. sulphurous acid, which, on evaporation, yields magnesia sulphite, $Mg\ SO_3$, which is not very soluble in distilled water. I then mix the sulphite of magnesia thus formed with distilled water, in the proportion of 16 grs. to oz. j. and pass into it sulphurous anhydride until a clear solution is obtained. The result is a solution of magnesia bisulphite.

The dose may be one tablespoonful, containing about nine grains of the salt; its action is a mild aperient antiseptic, preventing butyric fermentation in the stomach, etc. I have tried it myself, and on two other gentlemen, and, as far as I can judge, it has the desired effect.

Apiol.—The active principle of the seeds of Petroselinum Sativum, acts on the system very much the same as Quinine, producing in a dose of about fifteen grains, slight cerebral excitement, without any unpleasant effects of any kind. In large doses it produces headache, giddiness, morbid sight and sounds, with all the characteristic effects of a large dose of Quinine. Administered for intermittents, in temperate latitudes, eighty-six per cent. of cures have been reported. It acts slightly as a diuretic, and is said to have a sedative influence over the uterus.

UTERINE TONIC.—

R.—Macrotin, . . . . gr. 40.
Ferri sulph, . . . . gr. 60.
M.—Et fiat mass, div. pil. No. 40.
S.—One, two, or three times a day.

I wish to call the attention of the profession to the (Eclectic) remedy, Macrotin (described in King's Dispensatory), as a uterine tonic. Dr. Gallaway, of this place, first directed my attention to it, and I have found it a valuable remedy in all functional derangements of the uterus. I sometimes combine it with Sulph. Morphine in Dysmenorrhœa, when there is much pain ; and often use only the Macrotin in gr. j. doses two or three times a day ; but more often in the form above. Sometimes it causes pain in the head—then it will be well to discontinue the remedy a few days.

Its action is very slow, therefore of no use in labor (as *parturienet*). J. M. LEWIS, M. D.

THE RELATIONS OF ASTHMA, ANGINA PECTORIS, AND GASTRALGIA.—Dr. Anstie calls attention in the *British Medical Journal* for November 11, to the pathological and therapeutical relations which seem to unite these three diseases, making it evident, he thinks, that they depend on neurosis of the vagus, which is of central origin, and in the large majority of cases is mainly or entirely due to inherited peculiarities of the central nervous system. The evidence in favor of this view arranges itself under five heads. 1. Inferences from the known physiological functions of the vagus. 2. Evidence of the interchangeability of asthma, angina, and gastralgia in the same individual. 3. Evidence of the pathological connection of these neuroses with neuralgia of the fifth nerve. 4. Evidence of the common dependence of asthma, angina, gastralgia, and neuralgia of the fifth on peculiar inherited neurotic tendencies. 5. Evidence from the similarity of effects produced by certain remedies on all these maladies.

STRYCHNIA IN ALBUMINURIA.—Brignoli, in *Lo Sperimentale*, besides recommending nux vomica in various neuroses, gastralgia, dyspepsia, cardiac palpitations, periodic cough, etc., states that he had observed it to have a marked effect in retarding the progress of albuminuria, especially the scarlatinal form with anasacra. He cites twelve cases of complete recovery.—*British Med. Jour.*, Oct. 28, 1871.

DR. DEBOUT'S PILLS FOR MIGRAINE.—These pills are exceedingly recommended as having power to cure in many cases this obstinate affection.

| | | |
|---|---|---|
| R.—Quinia sulph., | . . | 3 grms. (46 gr.) |
| Digitalis (powdered) | . | 1 grm. 50 (23 gr.) |
| Syrup, | . . . | q. s. |

Mix, and divide into thirty pills. One to be taken every night at bed-time.—*L' Union Pharmoceutique*, Sept., 1871.—*New Remedies.*

CHLORAL FOR TOOTHACHE.—Dr. Page, in the *British Medical Journal*, recommends chloral hydrate as a local application in cases of toothache. A few grains of the solid hydrate introduced into the cavity of the tooth upon the point of a quill speedily dissolves there; and in the course of a few minutes, during which a not unpleasant warm sensation is experienced, the pain is either deadened, or more often effectually allayed. A second or third application may be resorted to if necessary.

IODURETTED GARGLE FOR SYPHALITIC ULCERATION OF THE MOUTH.—

| | | |
|---|---|---|
| R.—Iodide of potassium, | . . | 0.60 parts. |
| Tincture of iodine, | . . | 2.00 " |
| Distilled water, | . . . | 140.00 " |

M.—Dr. Gauthier, *Gironale italiano delle malatie veneree e della pelle*, Nov., 1870.—*New Remedies.*

BULIMIC DYSPEPSIA.—This form of dyspepsia is characterized by a feeling of emptiness of the stomach after eating; by diarrhœa occurring almost immediately after meals. Patients believe that they digest rapidly, and that their disease is intestinal. Where the diarrhœa is constant, the Laudanum of Sydenham given at first in *single drop* doses (before and not after eating) to be increased according to the demands of the case, and its control over the diarrhœa is said, by the late M. Trousseau, to be "wonderful" in its effects.

COLLODIUM - PAPER—COUNTER - IRRITATING COLLODIUM.—A very viscid and perfect collodium-paper may be prepared by the following formula:

| | | |
|---|---|---|
| R.—Sulphuric acid, : . | (1.82) | 200 parts. |
| Nitric " . . | (1.37) | 100 " |
| White filtering-paper, previously purified by hidrochloric acid, | | 10 " |

Mix in a glass vessel, and allow to stand about three hours till the reaction is complete. Then wash well. The paper thus obtained is freely soluble in a mixture of alcohol and ether, or if a little residue is left after solution it remains at the bottom; a very useful collodium for counter-irritation may be prepared according to the following formula:

No. 1. A very energetic preparation:—

| | |
|---|---|
| R.—Alcohol, . . . . | 3.50 parts. |
| Ether, . . . . | 11.50 " |
| Paper powder, . . . | 1.00 part. |

Mix, and add 10 parts of resin of thapsia, dissolved in 16 parts of alcohol.

No. 2. A weaker preparation—one more generally useful:—

| | |
|---|---|
| R.—Alcohol, . . . . | 12.00 parts. |
| Ether, . , . . | 45 " |
| Paper powder, . . . | 5 " |

Dissolve, and add: Resin of thapsia, 30 parts, dissolved in 63 parts of alcohol.—*L' Union Pharmaceutiqe*, October, 1871.—*New Remedies.*

CHOREA TREATED BY SULPHATE OF ZINC.—Dr. Dickinson (London Lancet) at Hospital for Sick Children, London, uses sulphate of zinc in chorea. In one case he says:

" She was put on two grains of sulphate of zinc in half an ounce of water, three times a day," " increased by the addition of a grain either every day or every other day, until on the twenty-fourth day, she had taken twenty-six grains three times a day. From the first she steadily improved, and by the time 19 or 20 grains had been reached, she was almost free from irregular movements. The quantity was then gradually reduced to 19 grains on the 31st day, and in four more days was discharged recovered."

GLANDULAR ENLARGEMENTS.—Dr. Carpenter (Oregon Medical & Surgical Reporter), states that he has found muriate of ammonia extremely beneficial in glandular enlargements.

Chloral.—Dr. S. W. Butler (Med. & Surg. Reporter), gives the following directions for giving this drug:

Chloral hydrate should not be prescribed in solution or mixture, excepting when it is to be used *immediately*. The proper way to administer it is as follows: Procure a number of small drachm vials, well corked—homœpathic vials answer the purpose very well—weigh out your doses of *pure chloral hydrate*, 10, 15, 20 or 30 grains, and put them into the vials, and securely cork them, and admininster them as required, by dissolving in a little sweetened water. *Never let it stand in a state of solution.* In ordinary cases, we should prefer the smaller doses mentioned above, repeated in fifteen minutes to half an hour, till the desired effect is produced.

Anti-neuralgic Powders.—(Langlebert.)—

R.—Powdered cubebs, 68 grammes, = oz, ij. dr. iss.
Carbonate of soda, 4 grammes, = dr. i.

Mix, and divide into 36 powders. Six to twelve daily for the neuralgic urethral pains which persist after the drying of a blennorrhagic discharge. In addition, use three injections of one or two minutes' duration of the following solution:

R.—Distilled water, . . . 100 parts.
Atropia sulph., . . 10 to 20 " M.

*Revue d Therap. Medico-Chirurg.—New Remedies.*

Treatment of Small Pox.—Dr. D. P. Boyer, in giving his method of treating variola, (Med. & Surg. Reporter), says:

"I gave a solution of 2 grains of carbolic acid, and 15 or 20 grains of sulphite of soda every three (3) hours *with no other* treatment than an ordinary purge during the initiative or forming fever. The result, after several months' trial with myself and son, has been that in *every case of variola* and confluent small-pox, on the fourth day of the eruption, the swelling of the face abated, the pulse fell to a normal rate, and the tongue commenced cleaning, the eruption commenced to dry up, and the pustules withered and *shrivelled.* By the seventh and eighth day of the eruption the patient was convalescent, without a sign or mark of having small-pox after the slight desquamation of the light scales, or scales fell off.

In no case by this treatment did the pustules positively mature, but always dried up before maturation. Externally any soothing or cooling application for the first three days is all that is required, to allay the itching, etc."

Bromide of Calcium.—Dr. Wm. A. Hammond, N. Y., (N. Y. Med. Jour.), writing of this drug, remarks:

"The dose is from fifteen to thirty grains or more for an adult. It is especially useful in those cases in which speedy action is desirable, as, owing to its instability, the bromine is readily set free, and its peculiar action on the organism obtained more promptly than when either of the other bromides is administered. Chief among these effects is its hypnotic influence, and hence the bromide of calcium is particularly beneficial in cases of delirium tremens, or in the insomnia resulting from intense mental labor or excitement.

Thus, I gave a gentleman, who, owing to business anxieties, had not slept for several nights, and who was in a state of great excitement, a single dose of thirty grains. He soon fell into a sound sleep, which lasted for seven hours. The next night, as he was wakeful, I gave him a like dose of bromide of potassium, but it was without effect, and he remained awake the whole night. The subsequent night he was as indisposed to sleep as he ever had been, but a dose of thirty grains of bromide of calcium gave him eight hours sound sleep, and he awoke refreshed, and with all unpleasant cerebral symptoms—pain, vertigo, and confusion of ideas—entirely gone.

In a number of other instances a single dose has sufficed to induce sleep, a result which very rarely follows the administration of one dose of any of the other bromides.

In those exhausted conditions of the nervous system attended with great irritability, such as are frequently met with in hysterical women, and which are indicated by headache, vertigo, insomnia, and a mental condition of extreme excitement, bromide of calcium has proved in my hands of decided service. Combined with the syrup of the lacto-phosphate of lime, it scarcely leaves anything to be desired. An eligible formula is—

℞.—Calcii bromidi, . . . . oz. i.
Syrup. lact. phos. calc., . . oz. iv.
M. ft. sol.

Dose, a teaspoonful three times a day in a little water.

Vomiting of Pregnancy.—Dr. Hubert, in a memoir read before the Medical Society of Lyons (*Lyon Medical*, 15th Oct. 1871, p. 476), produces evidence to show that in some cases the symptom depends upon misplacement and movement of the uterus, and that it may be arrested by the immobilization of the uterus by suitable bandages and the use of Hodge's or Zwanke's pessary.—*British Medical Journal*, Oct. 28, 1871.

Cod Liver Oil—Pepsine—Pancreatine.—Dr. Henry Gibbons (Pacific Med. & Surg. Jour.), speaking of cod liver oil, says:

"Very often the oil remains unchanged in the stomach, and continues for many hours to rise in the mouth. Here it not only fails to do good, but is positively injurious by retarding digestion and destroying what little appetite the patient may have had. A lack of pepsine may cause this difficulty—the office of pepsine in regard to oily substances being, as far as appears, to dissolve the albuminous cell-wall, leaving the oil to be acted on, after it has passed from the stomach, by the pancreatic secretion. It is easy to replace the deficiency in the gastric juice by the administration of pepsine. But there may be another link in the chain wanting. The fatty granules require to be acted on by the pancreatic secretion in order to perfect a series of processes by which the oil is assimilated and rendered remedial. The pancreatic secretion may be deficient; and here the want may be artificially supplied by the administration of pancreatine.

He then adds: "Pepsine is well known to the profession, having been in general use for many years. Pancreatine is of more recent introduction, and has not been so extensively employed. We believe it is not properly appreciated. In our hands it has certainly done much good in certain cases of indigestion, or perhaps it would be better to say, of uncertain and irregular digestion, especially in persons of morbid nervous sensibility. It is particularly valuable in feeble digestion attended with occasional diarrhœa—cases in which the diarrhœa appears to be excited by the accumulation of undigested food, and in which it alternates with constipation or intestinal lentor. Here a teaspoonful of Wine of Pepsine, taken immediately before eating, and a teaspoonful of Wine of Pancreatine half an hour or less after eating, will often be followed by the most gratifying results. Under this treatment anorexia will disappear and give place to appetite and enjoyment of food, and regular defecation will be established, so that the annoying exhibition of aperients and astringents may be enterely dispensed with."

Carminative Powder for Infants.—

| | | |
|---|---|---|
| ℞.—Fennel seed, . . . | 25.0 | grains. |
| Aniseed, . . . . . | 50.0 | " |
| Powdered sugar, . . | 350.0 | " |
| Powdered opium, . . | 1.0 | " |

Make into a powder, ten grains of which will contain .02 grains of opium.—*Zeitschrift des Osterreich. Apothek. Vereines*, Sept., 1871.—*New Remedies.*

Syphilis.—Dr. F. F. Maury, in speaking of the primary specific sore of syphilis (Med. & Surg. Reporter), says:

"*Never cauterize a primary specific sore with nitrate of silver; it is not a caustic, but simply a stimulant and alterant.* Use the mineral acids, such as the sulphuric and nitric, and the acid nitrate of mercury. For the chancroid the carbo-sulphuric paste is one of our best caustics; it stops the pus from spreading, and thus prevents auto-inoculation. This paste is made by taking of finely powdered willow charcoal and the strong, fuming, or Nordhausen, sulphuric acid, adding sufficient drops of the latter to reduce the former to the consistency of a paste. It must be made as wanted, for it will not keep. You apply this paste by spreading a quantity, of sufficient surface to cover the sore, upon a piece of lint or old soft muslin. If the chanchre is under the foreskin, retract the latter, apply the paste directly upon the sore, and then when the foreskin is drawn back, the lint comes between it and the paste, and thus prevents cauterization in a spot where it is not wished. This paste is not allowed to be scraped off, but must remain until it comes away with the eschar, which is generally in about a week.

Another preparation is Canquoin's paste, made by adding together two parts of dry chloride of zinc, four parts of flour, and one part of glycerine. This is rolled into a layer from $\frac{1}{6}$ to $\frac{1}{8}$ of an inch in thickness, and spread upon cloth; and thus when the caustic is applied, as in the previous case, it is prevented from coming in contact with anything but the sore. If the chloride of zinc deliquesces, which it is apt to do, it must be dried again.

These, then, are the caustics you must use—thorough agents, which do their work well. By cauterizing with nitrate of silver, we merely cover up the plague spot, not destroy it, and thus allow our patient to become a subject of syphilis!

If my lecture but succeeds in impressing this one point upon you, you will be well repaid for coming here this morning.

In speaking of mercury, he says:

" Another thing that you must rid your minds of is mercury in connection with primary syphilis. Remember, *you never give mercury with soft chancre; it but makes matters worse.* We give it in cases of hard chancre; not for its specific effect, but to promote, if possible, the absorption of the effused lymph.

Eulenberg's Formula for the Vomiting of Pregnancy.—

℞.—Tincture of iodine, . . . . 1 part.
Rectified alcohol, . . . . 12 " —M.

Three drops to be given many times a day.—*L' Union Medicale.*

Fistula in Ano.—In August, 1871, John Connor, who, for nearly eighteen months, had been suffering from a fistula in ano, applied to me for treatment, requesting a cure if possible without a surgical operation. I introduced a small probe of platina wire into the fistulous canal, and a wooden scoop into the rectum as a point d'appui for the extremity of the probe. During the operation a galvanic current of fifteen cells, steadily increased, was allowed to pass for fifteen minutes. The battery having been freshly filled that day, the probe became heated so rapidly that I was compelled to desist from the application sooner than I had wished. However, I was gratified to find that, at the expiration of five days, the fistula had entirely healed.—*Dr. Murray, in Med Rec.*

Fever Mixture.—The value of aconite in allaying fevers is apparently not so completely recognized by the profession as it ought to be, and having found the following recipes very useful in allaying fever, and in controlling it, when not dependent upon a deep-seated cause, I offer them here.—[Ed. N. R.]

R.—Tincture of aconite root, . gtt. xxiv.
Sweet spirits of nitre, . . f oz. ij.
Solution (or mixt.) of citrate of potash, f oz. iv.—M.
S. Tablespoonful every one, two, or three hours.

This formula, containing two drops of the tincture of aconite root to the dose, should be given cautiously, and every hour only in urgent cases which can be very carefully watched. Many women will scarcely bear it given every two hours. The following formula is more generally applicable and safer :

R.—Tincture of aconite root, . . gtt. xii.
Sweet spirits of nitre, . . f oz. ij.
Solution (or mixt.) of citrate of potash, f oz. iv.—M.
S. Tablespoonful every one or two hours.

Where there is much restlessness with the fever the following formula is very useful :

R.—Tincture of aconite root, . . gtt. xii.
Sweet spirit of nitre,
Comp. spirit of nitre, . aa f oz. iss.
Camphor water, . . . f oz. iij.
Morphiæ sulph., . . . gr. ss.—M.
S. Tablespoonful every one or two hours.—*New Remedies.*

Liebreich's Operation for Extraction of Cataract.—Prof. Liebreich says that during the four years past he has in more than three hundred cases employed the following method for extracting cataract in preference to the one recommended by Graefe, which he had formerly employed, and finds it to be, in many respects, its superior. The incision of the cornea is to be made with the smallest possible Graefe's knife in the following manner: "Puncture and contra-puncture are made in the sclerotic about one millimetre beyond the cornea, the whole of the remaining incision passing with a very slight curve through the cornea, so that the centre of it is about one millimetre and a half distant from the margin of the cornea. This incision can be made upwards or downwards, with or without iridectomy, and the lens can be removed through it with or without the capsule. If, as I now practise, the extraction is made downwards without iridectomy, the whole operation is reduced to the greatest simplicity, and does not require narcosis, assistance, elevator, or fixation; and only two instruments, Graefe's knife and one cysotome with Daviel's spoon. —*Br. Med. Jour.*, No. 570.

PARTIAL HEMIPLEGIA.—Prof. N. S. Davis, (Chicago Medical Examiner), says:

"The treatment, of course, is iodide of sodium, or potassium and bichloride of mercury. When there is pain, an anodyne may be added as in the formula below:

| | | |
|---|---|---|
| R.—Iodide of sodium, | . . . | dr. iij. |
| Bichloride of mercury, | . . | gr. j. |
| Fl. Ext. Conium, | . . . | oz. ss. |
| Simple syrup, | . . . . | oz. ss. |
| Aqua Men. Pip, | . . . | oz. iij.—M. |

S. One teaspoonful four times a day.

Under this treatment the pain is generally easier in four or five days, and in ten days gone. Then the dose may be reduced to three times a day for two or three weeks, and to twice a day for three or four months. Tonics will be required, both iron and the bitter barks, or the syrup of the hypophosphite; strychnia, gr. 1-40, and nitric acid, gtts. 1½, are tonics particularly adapted to these cases.

BILIARY CALCULI.—Prof. N. S. Davis (Chicago Medical Examiner, says:

"A Jewish lady had been subject to repeated attacks of severe pain, etc. Being called in the midst of one of the paroxysms, I was satisfied from the character and situation of the pains, and from the marked jaundice, that it was a case of biliary calculi. The nitric acid was ordered for her, 6 gtts. to be taken at each meal time, and continued for several weeks. She had one subsequent attack, a short time after commencing treatment, but has had none since.

I can recollect three other cases where the re-formation of calculi has been entirely prevented. In two of the cases an opposite course of treatment was tried, an alkali, liquor potass, being given instead of the acid.

Our aim in the treatment of these cases should be, first, to relieve temporarily the pain, by narcotic, warm fomentations, etc.; and secondly, to so change the constitution of the bile as to prevent the formation of the new calculi. Their formation is probably caused by an excess of chloresterine in the bile, which becomes crystallized. The calculi formed are sometimes so large as never to pass through the ducts; but being retained, and blocking up the ducts, they produce permanent derangement, congestion, atropy, jaundice, etc.

The treatment by ether, acids, or alkalies seems to accomplish the desired change in the constitution of the bile."

Vaginismus.—M. Gueneau de Mussy, in the *Gazette des Hopitaux*, June, 1871, commends the treatment of this obstinate affection by the following formula:

| | | | |
|---|---|---|---|
| R.—Butter of cacao, | 20 | grammes. | (306.8 grs.) |
| Bromide of potassium, | 3 | " | (46.0 grs.) |
| Ext. of belladonna, | 1 | " | (15.34 gr.) |

Make into ten vaginal suppositories. One to be used each night for a week.

Some drops of the following formula are also to be given hypodermically:

| | | | |
|---|---|---|---|
| R.—Muriate of morphia, | 50 | centigr. | (7.57 grs.) |
| Sulphate of atropine, | 1 | " | (.1534 gr.) |
| Distilled water, . | 10 | grammes. | (153.4 grs.) |

Mix and make a solution.

If pruritus exist with the vaginismus, the suppositories must be used for a longer time, and arseniate of soda be exhibited.—*New Remedics.*

Tetanus.—Among other interesting papers lately read before the Academy of Sciences in Paris, was one by M. Demarquay, in which he showed that several cases of lock-jaw had been cured by extremely hot-air baths, followed by the injection of morphia under the skin.—*The Lancet.*

OBSERVATIONS ON RACHITIS.—The last physical sign to which attention will be directed in connection with the head is dentition. This is ordinarily retarded, and the simple fact that a child has reached his ninth month without dentition having commenced, is enough to lead an intelligent physician to suspect commencing rachitis. Not only does dentition commence late and progress slowly, but the eruption of the teeth is attended with great pain, and they are irregular in time of appearance and position.

The extent to which dentition may be delayed varies much in different cases. It is no uncommon thing to meet with rickety children a year or eighteen months old in whom dentition has not commenced. The writer has met with a single instance of a rickety child in whom the primary teeth failed to make their appearance at all. The same was noticed by Van Swieten, for he says, "I have seen some youths, who had been negligently treated in the beginning of this disease, continue toothless during life."*

They appear with difficulty, and often with severe suffering, and are irregularly set in the jaws. This is owing to the imperfect development of the jaws, which, being smaller than in healthy children, do not afford sufficient room for the natural eruption of the teeth. To this cause may be ascribed many of those symptoms which authors so often attribute to dentition. In a healthy child this, which is a natural and physiological process, is unattended with any serious phenomena, and it is in these sickly and cachectic infants that the teeth appear painfully, slowly, or not at all. Moreover, the cachexia must be peculiar—in other words, rachetic—or this will not occur, for in cases of chronic diarrhœa and other wasting diseases of children, the teeth appear at the usual time and in the usual manner. This is true, though the little patient may be emaciated to the last degree, while in rickets dentition is often much delayed and very difficult when the lesions connected with the long bones are but slight and the child presents the external evidences of moderate health.

The teeth themselves are imperfectly developed, deficient in enamel, decay in a very short time, and, as Glisson so truthfully remarks, "fall out by pieces." It is no rare thing to see one of these children almost toothless long before the permanent set should appear.—*Dr. John S. Parry, in Am. Jour of Medical Sciences.*

HOW TO PREVENT THE RUSTING OF STEEL INSTRUMENTS.—A correspondent confidently recommends equal parts of carbolic acid and olive oil, smeared over the surface of the instruments, as

* Commentaries, etc., Edinburgh, 1776, vol. xvii. p. 385.

an unfailing preventive of rust in any climate. He states that it is a plan much used by medical officers in the navy, and it is found to preserve the burnish and brightness of the steel, however moist and warm the atmosphere may be. Our correspondent adds that it will also be found advantageous to have the fittings of instrument-cases made only of brass and polished wood, as the glue with which the velvet usually employed is fastened on is apt to absorb moisture and remain damp for a considerable time.—*The Lancet.*

Drops for Gastralgia (Dr. Gallard's Formula).—

| | | |
|---|---|---|
| R.—Distilled water of cherry laurel, | | 5.00 parts. |
| Muriate of morphia, | . . | 0.10 " |

Mix and dissolve.

One drop on a lump of sugar immediately before meals.—*Union Medicale.—New Remedies.*

Foot and Mouth Disease in Children.—Some weeks since we stated, on the authority of the Chairman of the Hartford Chamber of Agriculture, that children fed on the milk from diseased cows had suffered from a complaint similar to the "mouth disease." Since then Dr. Alfred Packman, of Puckeridge, has treated several cases of this complaint, and states in a certificate that the patient "suffered from a peculiar eruption of the mouth, nose and face, accompanied by sore tongue and throat, and salivation. I have no sort of doubt that the affection was caused by drinking milk from cows suffering from foot and mouth disease." —*Medical Times and Gaz.*

Carbolized Gargle for Diptheria.—Charles Sedgwick, Jr. (New Remedies) recommends the subjoined formula:

| | | |
|---|---|---|
| R.—Carbolic acid, | . . . . | 20 m. |
| Acetic acid, | . . . . | dr. ss. |
| Honey, tincture of myrrh, each, | . | f dr. ij. |
| Water, to | . . . . | f oz. vj. |

The acids to be shaken together well before the other ingredients are added.—*Medical Record.*

Tincture of Capsicum as a Styptic.—Among the multitude of known styptics used to stop excessive bleeding after tooth extraction, I am not aware if every dentist has tried the effect of two or three drops of tinctura capsici on wool, firmly pressed into the cavity; my experience goes to prove it the best remedy extant; one application generally answering the purpose.—*John Branson, Brit. Jour. of Dental Science.*

Ulcers of the Leg.—In the treatment of ulcers in the lower extremeties, I have almost invariably applied the positive pole of the battery, through a medium of a disk of platina, silver, or nickel, to the ulcer itself, closing the circuit, and by means of the wet chamois electrode close to and around the diseased part. The disk I have used is about one-eighth to one-twelfth of an inch in thickness. I cannot recommend pure nickel for this purpose, as it is difficult to clean and polish it as needed before it can be used again. Platina is decidedly preferable, as it is the cleanest and best metal.

A man, aged 30 years, with seven ulcers upon the lower third of the right leg, applied to me for treatment during the month of October instant. These ulcers were of two years' standing, and as the case appeared to me to be one favorable for testing the relative merits of the poles, the size and condition of each ulcer being as nearly as possible uniform, I selected four for treatment by the positive and three by the negative pole. I first cleansed the sores with a piece of sponge wet with a weak solution of chlorinated soda, and having *partially* dried them, I applied the flat-disk electrode as before explained. The superiority of the flat-disk over the button or ball-shaped electrode is evident, when we consider that ulcers heal from the sides first rather than from the bottom. I applied the galvanic current as strong as could be borne to each ulcer for the space of five minutes, occasionally wiping away any discharge. This treatment was repeated every third day. In two weeks the healing process, in the four under the positive pole, was considerably in advance of that of the three under that of the negative. In general I do not limit myself to any specified time in the galvanization of ulcers, but continue the application of the current until they present the *appearance of having been slightly seared with the actual cautery.—Dr. Murray, in Med. Record.*

A Remedy for Freckles.—

| | | |
|---|---|---|
| R.—Sulpho-carbolate of zinc, | . | 2 parts. |
| Glycerine, | . . . . | 25 " |
| Rose-water, | . . . . | 25 " |
| Spirits, | . . . . . | 5 " |

Dissolve and mix.

The freckled skin is to be annointed with this twice daily, the ointment being allowed to stay on for one-half to one hour, and then washed off with cold water. Anæmic persons should also take a mild ferruginous tonic. In the sunlight a dark veil should be worn.—*Zeitschrift des Allegem. Oester. Apothek. Vereines*, October 1, 1871.

Deficient Secretion.—The following is a short epitome of nine cases of deficient secretion of milk, in which local faradization was applied directly to the mammary gland.

In four the electro-magnetic current was applied, with negative results.

In two the magneto-electric current was applied. In one of these the result was negative ; in the other success attended after daily application for sixteen days.

In three, the magneto-electric current was also applied, with the result of producing a good supply of the lacteal fluid so long as the use of the current was repeated daily ; but the secretion ceased if it was omitted for twenty-four hours.

One woman, to whose breast I applied the galvanic current, gave the breast to her child immediately after. In less than an hour the child was attacked with severe vomiting, purging, and convulsions, although it had been previously remarkably healthy. I attributed the sickness to an electrolytic change in the milk, the effect of the galvanic current.—*Dr. Murray in Med. Record.*

Anæsthetic Calcareo-Glycerine for Burns, &c.—Dr. De-Bruyne extols the following liniment for burns, &c. :

R.—Freshly precipitated hydrate of lime, grammes iij.
Glycerine, . . . . " cl.
Mix, and heat moderately, and then add :
Chlorinated chlorohydric ether, grammes iij.

The liquid thus obtained is transparent and clear. A compress of fine linen is thoroughly wet with this and applied on the burnt part. Immediately over this is to be placed a piece of gold-beater's skin, impermeable taffeta, or even flannel, so as to secure complete occlusion, and prevent the evaporation of the liniment.

Dr. B. is convinced of the beneficial effects of this dressing, not only in burns, but also in ill-conditioned wounds ; in atonic, callous, fungous, and foul ulcers ; and likewise in certain cutaneous diseases, especially such as are dry and squamous' accompanied with pruritus.—*Revue de Therapeutique*, Oct. 15, 1871., from *Journal de Bruxelles*, Jan. 1871.—*Am. Jour. Med. Sciences.*

Strychnia in Albuminuria.—Brignoli (British Medical Journal, October 28 ; from La Sperimentale), besides recommending nux vomica in various neuroses, gastralgia, dyspepsia, cardiac palpitations, periodic cough, etc., states that he has observed it to have a marked effect in retarding the progress of albuminuria, especially the scarlatinal form with anasacra. He cites twelve cases of complete recovery.

Gallic Acid in Uterine Hemorrhage.—In a late session of Gynæcological Society of Boston (*Jour. Gynæcological Soc.*), Dr. Hazelton remarked that in a case of flooding from retained placenta, after abortion, he had relied on good nourishing diet, with lead and opium. After the cervix had dilated sufficiently, he removed the mass with an ordinary polypus forceps.

Dr. Weston advocated the use of gallic acid. Dr. Martin had read Dr. Simpson's paper on this remedy, and had given it a fair trial. According to his observation, the patient was required to take from one to two drachms before any effect was perceptible.

Cure for Toothache.—Dr. Henry T. Reynolds, of Baltimore, writes to us that "for eighteen months I have been using acetate of lead as a remedy for what Burns calls the "hell of all diseases" —toothache. I find it to act better than any of the numerous remedies proposed in the books, and in cases in which it is applicable, the relief is instantaneous. Let the patient apply from one to three grains to the cavity for a moment or two, then spit it out. It fails in fewer cases than any remedy that I have tried—not more than eight per cent."—*Med. News and Richmond and Louisville Med. Jour.*

Drastic Pills for Dropsy.—

| | | | |
|---|---|---|---|
| R.—Elaterium, . | 10 | centigr. | (1.5 grains.) |
| Extr. of hyosyamus, | 60 | " | (9 grains.) |
| Extract of gentian, | 60 | " | (9 grains.) |

Mix and make into 10 pills.

One or two to be taken in dropsy, when it is desired to rapidly run off water by the bowels, and if necessary the dose may be increased to three.—*L' Union Medicale.*

Popliteal Aneurism Cured by Flexion in Three Days.—The *Bulletin de l'Academie Royale de Medicine de Belgique* (British Medical Journal, December 23) contains an account by Dr. Larondelle of the cure of a popliteal aneurism of the size of an orange. Dr. Larondelle adopted Mr. Ernest Hart's method of forced flexion of the leg upon the thigh. There was œdema of the foot and leg. The bandage employed was applied after the fashion recommended by Mr. Hart in his first paper in the Medico-Chirurgical Transactions ; and the patient, as in his second published case, was allowed to walk about the room with the help of a crutch. The bandage was solidified by starch. The flexion seems to have been forced a little in excess. At the end of the second day, as the patient was complaining much of the pain, the bandage was removed. A second bandage was applied, and on the third day the tumor was found to be solidified. The cure thus effected was permanent; and the tumor, at the end of five months, was reduced to the dimensions of a small, hard kernel.—*Phil. Med. Times*, Feb. 15, 1872.

Ipecacuanha Given in Enema for Dysentery.—Dr. Charles Moore Jessop extols (Indiana Medical Gazette, July, 1871) the efficacy of ipecacuanha given in enema for the cure of dysentery. He gives ten grains of the powder with half a drachm of laudanum in two ounces of decoction of arrowroot or mucilage, three times a day; when the symptoms abate, twice a day, and, finally, once a day.

In the No. of the same journal for Sept., Surgeon J. F. Baxter relates the following case confirmatory of Dr. Jessop's statement:

"A young married lady who had suffered from a recent attack of acute dysentery, having had one or two relapses in the space of a few months, came under my observation in the autumn of last year; she was then passing small quantities of blood five or six times daily, but declined to resort to medicine; towards the year, however, she had another sharp attack, and I was asked to see her.

"I prescribed five grains of ipecacuanha, with a small quantity of opium, in pills, which were shortly afterwards rejected by the stomach. I reduced the quantity to two grains, with the same result. I then tried every remedy I could think of, with a view to make the stomach tolerant of the drug, but without success.

"The patient suffered great pain, was constantly on the move to and from the bath-room, was tormented with persistent and severe straining, and as nothing remained on the stomach, I felt it not unlikely that I should lose her; the thought, however, occurred to me to administer ipecacuanha in enemas, and accordingly I gave two scruples of ipecacuanha wine, fifteen minims of tincture of opium, and one ounce of thin arrowroot in enema. This gave her relief and sleep for about four hours—the first she had for several days. I continued the treatment, and the following day she was considerably improved, and in four days more she was quite free from pain, purging, or blood; she rapidly recovered, and although she had a slight relapse some months afterwards, I have since heard that she was quite well."—*Am. Jour. Med. Sciences.*

Croup, catarrh, and colds in children can be cured by giving equal parts of the syrup of sanguinaria and cod-liver oil.

SULPHATE OF IRON AS A LOCAL APPLICATION IN PHLEGMASIA DOLENS.—Dr. R. W. Creighton speaks favorably (British Med. Journal, Oct. 21, 1871) of the efficacy of sulphate of iron as an external application in phlegmasia dolens.

"In recent cases," he says, "it is best employed in the form of lotion (twenty to thirty grains to an ounce of water), applied as hot as the patient can comfortably bear it. Much trouble is avoided, and the constant saturation of the skin with the lotion secured, by using spongio-piline, fastened loosely by tapes round the limb.

"All the cases which I have so treated—five or six in number—have made good and rapid recoveries; and in one only, which was not seen for several days after the disease commenced, was there any great amount of hardness left in the superficial venous trunks at the end of ten or twelve days. In this case, the application of equal parts of belladonna and iodide of potassium ointments was effectual in dispersing it. In all the cases, after the bowels had been freely acted on, the muriated tincture of iron, either alone or combined with quinia, was given internally.

"The rapid introduction of waste material into the blood during the puerperal state affords, I believe, some explanation of the action of sulphate of iron externally, and of the muriated tincture internally, in cases of phlegmasia dolens."—*Am. Jour. Med. Sciences.*

CATARRHUS VESICÆ.—The following relieved an obstinate case in two weeks:

| | | | | |
|---|---|---|---|---|
| R.—Fluid Extract Buchu, | . | | | |
| " " Uva Ursi, | . | *aa* | oz. ss. | |
| " " Gelseminum | . | | dr. i.—M. | |

S. 50 drops 5 times daily.

CARBONATE OF AMMONIA IN PNEUMONITIS.—Dr. Nelson Nivison, of Geneva, N. Y. (National Med. Jour.), has long been in the habit of giving carbonate of ammonia in pneumonitis, and in nearly all cases of embarrassed pulmonary circulation. When this occurs from hepatization of the lungs there is no remedy so effectual. Whether it operates by stimulating the nerve-centres, and through them the innervation of the respiratory organs, or whether it modifies the chemical or vital properties of the blood, or produces both effects simultaneously, we may not, he says, in the present state of our knowledge, be able to determine. Clinical observation has most strongly impressed upon his mind the conviction that in some way it very efficiently aids in the resolution and absorption of the fibrinous blood constituents, which by their coagulation solidify the parenchymatous structures of the lungs

CURE OF HYDROCELE BY THE SETON.—This method is a modification of what is known as Pott's treatment, and has been lately recommended, especially by Mr. Henry Smith, an English surgeon, as perfectly safe, convenient, and effectual, patients being able to go about and even attend to their various occupations during the progress of cure; two cases only, out of thirty operated upon by him, suffered subsequently even of inflammation—one of these the result of imprudence in exercise—and no return of the disease in any. The operation is described by Mr. Smith as follows: Simply to puncture the tumor with a common suture needle, armed with a single thread, and, having brought the thread out a distance of one or two inches from the point of entrance, it is disengaged from the needle, and the two ends are tied lightly together. He further advises that this thread, as a rule, may be left in from eight to ten days, and at any time, if the inflammation seems inclined to fall short of the degree required, it may be increased by moving the thread.

Thomas R. Dupuis, M. D., Odessa, Ont. (The Canada Lancet), having had some experience in this treatment, is inclined to think that there is more risk in this treatment than by the injection of a stimulating fluid, which is quickly brought into contact with the interior of an already empty sac, which subjects all parts at once to the same grade of excited action, and which does not interfere with the tissues *external* to the parts acted upon.

VAN DEN COURT'S PILLS FOR CHORDEE.—

| | | |
|---|---|---|
| R.—Extract of belladonna, | . . | gr. ij. |
| Lupuline, | . . . . | grs. xij. |
| Camphor, | . . . . | gr. xij. |

Mix and make into eight pills.

S. Take from two to four at night.—*London Lancet.*

THE TEASPOON AS A MEASURE.—A writer in the *Canadian Pharmaceutical Journal,* who has examined the subject critically, says that the teaspoons have been gradually growing larger of late years, the spoon of the last century having been only about two-thirds of the size of that now in common use. He adds, however, that three sizes are made at the present time—large, medium, and small, containing 95, 85, and 60 minims respectively. Table-spoons, also, have increased, and vary from 4,5 to 6 fluid drachms in capacity. He infers that the dose of certain articles may be unsafe if a teaspoonful or a table-spoonful be ordered, and proposes to abolish the desert-spoon as a measure, substituting two teaspoonfuls.

Subcutaneous Injections of Ergotin in Uterine Affections.—The following cases are translated from the German of Swiderski by C. W. Stevens, M. D., of Charlestown, Mass.

The standard solution used in the cases I am about to mention is the following:

| | |
|---|---|
| R.—Ext. secale cornuti aquosi, . | dr. ss. |
| Spr. vini. rectif., . . . | dr. j. |
| Glycerine, . . . . . | dr. ii. |

The dose of this solution is from 12 to 18 minims or from 2 to 3 grains of ergotin, and acts usually in half an hour.

Case 1. A woman, aged 44, who had suffered for five years from menorrhagia. The uterus was greatly enlarged, hard on its posterior aspect, not painful, and with some retroversion. The anterior aspect was hard and thickened. The first subcutaneous injection of 12 minims or 2 grains was on the fifth day of profuse menstruation. In two hours uterine pains began, which lasted six hours, during which she passed many clots. On the ensuing day 3 grains were injected, and in less than two hours pains came on which lasted ten hours. On the next day there was no hemmorrhage.

Case 2. A woman of fifty, who has had menorrhagia for 8 years. The uterus was enlarged and perceptible above the pubes. After two injections the hemorrhage ceased, and the uterus descended so as not to be felt.

Case 3. A lady of 42 was having dangerous menorrhagia. An injection of 2½ grains brought on great pains and diminished the flow. On the following day another injection stopped the hemorrhage.

Case 4. A woman of 19, in her third month of pregnancy, began suddenly to have fearful metrorrhagia which ordinary means failed to arrest. An injection of three grains soon caused pains, when she expelled the fœtus and ended the flooding.

Case 5. A woman flowed ten weeks after a miscarriage, during which time she took ergot internally without avail. An injection, then, of three grains caused the flowing entirely to cease.

In two cases of *post partum* hemorrhage, after the failure of the usual methods of hemostasis, an injection of three grains arrested the flowing in a short time.

The same injections have been found very valuable in chronic metritis and hypertrophy, as well as in abnormal positions of the uterus, if not of too long standing. In these cases the injections are a little weaker, and are repeated every two or three days.

Therapeutical Use of Water.—Dr. T. C. Smith in the Chicago Medical Journal, with reference to the use of water, says:

A few cases to clinically illustrate the advantages of this treatment, and I will close.

Mr. J., æt 30, laborer; bilious temperament, complexion sallow, average height and build, pulse 70, of moderate fullness and force, habits good, skin dry, slightly above natural heat, urine and perspiration rather scant, costive, has constant feeling of dizziness and weight, with pain in the head, the latter often severe, tongue covered with thick yellowish fur, taste bitterish, has a feeling of hebetude all the time.

R. Mistura creta dr. j. ter die, as a placebo, and directed that he should drink all the water he could *between meals*, and report again in one week. He did so, and expressed himself as feeling better than for months. The treatment was continued for several weeks with good success, all unpleasant symptoms being dissipated. Since that time (two months) he has been in perfect health, and thinks he is so on account of the water cure.

In this case, molecular waste and elimination were retarded.—Water increased these processes by constituting a vehicle to carry out oxidized material floating in the fluids, and by causing increase of all the secretions, thus giving better opportunity for healthy metamorphosis.

Carminative Mixture.—

R.—Oil of camphor,
Oil of cajeput, . . *aa* f dr. ij.
Oil of cinnamon (or cloves), . f dr. j.—M.

Ten to fifteen drops on sugar for an adult.—*New Remedies.*

Uva Ursi as an Emmenagogue.—At a late meeting of the Gynæcological Society of Boston, Dr. H. R. Storer (Journal Gynæcological Soc.), in a discussion on Dr. Hjaltelin's monograph on "The Gynæcology of Iceland," stated that the use of uva ursi as an emmenagogue or oxytocic was not a familiar one to himself. Dr. Wheeler had heard of its employment, but could not recollect by whom. Dr. Warren thought it had been employed by the late Dr. Josiah Noyes, of Needham, who used it like secale cornutum.

Cystalgia.—In a case of a child one year old affected severely with Cystodynia, one drop of the Fluid Extract Gelseminum gave not only relief, but a cure in two days.

ETHER SPRAY IN DIAGNOSIS.—Dr. Richardson, at the Medical Society of London, recently explained a new application of ether spray, which he had for over two years past employed—viz : for the purpose of diagnosis in some forms of nervous disorder. He had noticed from the first use of the spray that the period required for producing freezing of the surface of the body varies with the condition of the patient, the weak and the aged being much more easily influenced than the robust. He therefore has been led to the practice of using the spray as a test of vascular tonicity of parts of the body, and inferentially of the nervous control over the vessels. In the case which had led to these observations—a case of obscure paralysis, brought before the Medical Society by Dr. Alfred Carpenter, of Croydon, on which a committee consisting of Drs. Richardson, Lockhart Clarke, Broadbent, Hughlings-Jackson, Carpenter, and Mr. Brudenell Carter had drawn up a report for the Society—this method of employing ether spray had been adopted, with the effect of finding that in certain of the paralyzed parts the freezing of the tissues could be established in from two to three seconds, while in a healthy subject, and in the same subject on other parts of the body, from eight to nine seconds of time were required, the ether used and the external conditions being the same. The practice is suggestive of extensive application in estimating what, in the absence of a better term, we may still venture to call "the vital power" of different individuals or of the same individual, generally and locally, under various conditions of disease.—*Med. Times and Gaz.*

FORMULA FOR CYSTITIS.—The following is used by Prof. Gross to relieve the irritation of the bladder produced by the presence of a stone:

R.—Uvæ Ursi, . . . . oz. j.
Sodæ Bicarb, . . . . dr. iij.—M.

S. Add to one and a half pints of boiling water, and take a wineglassful two or three times daily.—*Phil. Med. Times.*

